# Formulation and Stability of Ataluren Eye Drop Oily Solution for Aniridia

**DOI:** 10.3390/pharmaceutics13010007

**Published:** 2020-12-22

**Authors:** Celia Djayet, Dominique Bremond-Gignac, Justine Touchard, Philippe-Henri Secretan, Fabrice Vidal, Matthieu P. Robert, Alejandra Daruich, Salvatore Cisternino, Joël Schlatter

**Affiliations:** 1 Assistance Publique-Hôpitaux de Paris, AP-HP, Hôpital Universitaire Necker-Enfants Malades, Service de Pharmacie, Université de Paris, 75015 Paris, France; celia.djayet@aphp.fr (C.D.); justine.touchard@aphp.fr (J.T.); phsecretan@yahoo.fr (P.-H.S.); fabrice.vidal@aphp.fr (F.V.); salvatore.cisternino@aphp.fr (S.C.); 2 Assistance Publique-Hôpitaux de Paris, AP-HP, Hôpital Universitaire Necker-Enfants Malades, Service d’ophtalmologie, CRMR OPHTARA, Université de Paris, 75015 Paris, France; dominique.bremond@aphp.fr (D.B.-G.); matthieu.robert@aphp.fr (M.P.R.); alejandra.daruich-matet@aphp.fr (A.D.); 3 INSERM UMRS 1138, Team 17, Centre de Recherche des Cordeliers, Université Sorbonne Paris Cité, 75006 Paris, France; 4 Matériaux et Santé, Université Paris-Saclay, 92296 Chatenay-Malabry, France; 5 INSERM UMRS 1144, Optimisation Thérapeutique en Neuropsychopharmacologie, Faculté de Pharmacie, Université de Paris, 75006 Paris, France

**Keywords:** aniridia, ataluren, ophthalmic solution, rare disease, stability

## Abstract

Congenital aniridia is a rare and severe panocular disease characterized by a complete or partial iris defect clinically detectable at birth. The most common form of aniridia occurring in around 90% of cases is caused by PAX6 haploinsufficiency. The phenotype includes ptosis, nystagmus, corneal limbal insufficiency, glaucoma, cataract, optic nerve, and foveal hypoplasia. Ataluren eye drops aim to restore ocular surface PAX6 haploinsufficiency in aniridia-related keratopathy (ARK). However, there are currently no available forms of the ophthalmic solution. The objective of this study was to assess the physicochemical and microbiological stability of ataluren 1% eye drop in preservative-free low-density polyethylene (LDPE) bottle with an innovative insert that maintains sterility after opening. Because ataluren is a strongly lipophilic compound, the formulation is complex and involves a strategy based on co-solvents in an aqueous phase or an oily formulation capable of totally dissolving the active ingredient. The visual aspect, ataluren quantification by a stability-indicating chromatographic method, and microbiological sterility were analyzed. The oily formulation in castor oil and DMSO (10%) better protects ataluren hydrolysis and oxidative degradation and permits its complete solubilization. Throughout the 60 days period, the oily solution in the LDPE bottle remained clear without any precipitation or color modification, and no drug loss and no microbial development were detected. The demonstrated physical and microbiological stability of ataluren 1% eye drop formulation at 22–25 °C might facilitate clinical research in aniridia.

## 1. Introduction

Congenital aniridia is a rare and severe ocular disease. This panocular disease is characterized by a complete or partial iris defect clinically detectable at birth [1,2,3]. The disease is commonly associated with nystagmus, low vision, ptosis, corneal limbal insufficiency, glaucoma, cataract, optic nerve, and foveal hypoplasia. Congenital aniridia affects equally males and females and has a prevalence of 1:100,000 to 1:40,000 [1]. The most common form of aniridia occurring in around 90% of cases is caused by PAX6 haploinsufficiency due to intragenic mutation or chromosomal rearrangement in the PAX6 gene at 11p13. An autosomal dominant transmission is present in up to 90% of cases. Sporadic congenital aniridia may consist of 13% to 33% of cases as family forms consist of around two-third of cases. Congenital aniridia may be part of a syndrome as in WAGR contiguous gene syndrome (Wilms tumor, aniridia, genitourinary anomalies, and mental retardation) or in the rare Gillespie syndrome (cerebellar ataxia and mental retardation) [1,2,3]. A minority of different gene mutations may also be observed in congenital aniridia due to other gene anomalies [1,4]. The visual prognosis of aniridia is severe, with congenital low vision due to foveal hypoplasia and occasionally optic nerve hypoplasia. The severe evolution results from keratopathy associated with corneal opacification, glaucoma, and cataract [1,2,3,4,5,6]. In the corneal limbus, the loss of the stem cell niche in Vogt’s palisades progresses and causes corneal opacity called aniridia-related keratopathy (ARK), which can finally lead to blindness [7]. Therefore, a new approach for aniridia treatment has been proposed involving nonsense mutation suppression therapies, such as ataluren, which could limit aniridia disease progression and corneal damage [8,9,10]. As a result of the study, a phase 2 clinical trial was designed to evaluate the effect of oral ataluren in participants with nonsense mutation aniridia [11].

Ataluren, chemically known as 3-(5-(2-fluorophenyl)-1,2,4-oxadiazol-3-yl)benzoic acid, is a drug having nonsense codon suppression activity approved by the FDA and European agencies for the treatment of Duchenne muscular dystrophy in ambulatory patients aged 2 years and older [12]. Ataluren enables ribosomal read-through of mRNA containing such as a premature stop codon, resulting in the production of a full-length protein [13,14]. Currently, ataluren granules for oral suspension (Translarna^®^ 125 mg, 250 mg, and 1000 mg, PTC Therapeutics International Limited, Dublin, Ireland) are the only marketed form.

The formulation of an ataluren eye drops solution could be advantageous for the repositioning of ataluren in the ocular treatment of aniridia. Eye drops allow more effective corneal exposure while limiting systemic body exposure. Ataluren is a small and lipophilic molecule soluble in organic solvents, such as dimethylsulfoxide (DMSO), and very slightly soluble in water [15]. Ataluren (1% *m*/*v*) suspension in 0.9% saline vehicle containing 1% tween 80 as a co-solvent, and 1% carboxymethylcellulose to increase viscosity, also known as the ‘START’ formulation, was shown to rescue the corneal deficit in Pax6-deficient mice model of aniridia [10]. Although this preclinical study suggests a benefit of the topical administration of ataluren, its chemical stability over time, as well as its sterility, were not assessed to our knowledge [10]. The objective of our study was to develop a 1% ataluren solution free of particles and chemically and microbiologically stable at least over 2 months when stored at 25 ± 3 °C.

## 2. Materials and Methods

### 2.1. Chemicals and Materials

The pharmaceutical ingredient of ataluren was obtained from Sigma-Aldrich (St. Quentin Fallavier, France). DMSO USP grade was provided from Wak-Chemie Medical GmbH (Cryosure, Steinbach, Germany). Pharmaceutical grade castor oil was provided from Cooper (Melun, France). Pharmaceutical hydroxypropylcellulose and tween 80 were provided from Inresa-Pharma (Bartenheim, France). Other chemicals were of analytical grade. Titanium dioxide (99.5% Aeroxide^®^ P25, nanopowder, average primary particle size 21 nm) came from Sigma Aldrich (St. Quentin Fallavier, France). All solvents used were HPLC grade from Merck (Darmstadt, Germany). Cationorm^®^ was obtained from Santen (Evry, France) and contained mineral oils, cetalkonium chloride, tyloxapol, poloxamer 188, glycerin, buffer system (Tris-HCl/trometamine), and water for injection. The sterile preservative-free low-density polyethylene (LDPE) multidose opaque eyedropper Novelia^®^ was obtained from Nemera (La Verpillere, France) and distributed by CAT laboratory (Montereau, France). It was chosen for its capacity to maintain sterility in normal use and under conditions of misuse and extended use, including an anti-return valve system with a silicone membrane.

### 2.2. Formulation Development Assay and Preparation of Ataluren Eye Drops

Aqueous formulation of ataluren (1%) compounded with 0.9% sodium chloride, 1% tween 80, 1% ataluren (*m*/*v*), and 1% hydroxypropylcellulose (STAR) was prepared. This ataluren suspension was stored at 25 ± 3 °C, and the chemical stability was assessed during 21 days.

Other experiments were carried out by dissolving ataluren (1%) in pure castor oil and in a ready-to-use ophthalmic Cationorm^®^ solution, also known as hydrating and lubricating eye drops. Although the ophthalmic solution obtained was limpid, precipitates were formed in less than 3 weeks without the possibility of resuspension/dissolution despite manual shaking (data not shown).

Alternative ataluren (1% *m*/*v*) eye drops formulations using DMSO as co-solvent in castor oil were evaluated [16]. Ataluren 1% (*m*/*v*) compounded with 10% (*v*/*v*) DMSO and 90% (*v*/*v*) castor oil allowed its complete solubilization. First, ataluren powder should be mixed in DMSO until completely dissolved. Second, castor oil should be added, and the resulting oily solution was mixed by inversion for 1 min. The obtained ataluren solution was filtered through a 0.22 µm polyethersulfone filter (Millex, Merck Millipore, Fontenay-sous-Bois, France) and then sterilely distributed into the eyedropper vials (10 mL per unit) in a vertical laminar B-class airflow hood of the microbiological safety cabinet.

### 2.3. Analyses Performed on the Ataluren Formulations

#### 2.3.1. Visual Inspection

At each sample time, a visual inspection of the eye drop solution was made by the same operator, looking for a change in coloring, particles, or precipitate, compared with a control consisting of castor oil.

#### 2.3.2. Instrumentation

For each unit, ataluren was quantified using the liquid chromatography (HPLC) method adapted from the method described by Kong et al. [17]. Analyses were performed on a Thermo Scientific Ultimate 3000 chromatogram system (Villebon-sur-Yvette, France), including a quaternary pump (LPG 3400A), an automatic sampler (WPS 3000TSL), a diode array detector (DAD 3000RS) with 5 cm flow cell, and the associated software used to record and interpret chromatograms (Chromeleon^®^, Version 7.2.8, Thermo Scientific, 2018). The stationary phase consisted of a Kinetex^®^ C18 column (250 × 4.6 mm; 5 µm, Phenomenex, Le Pecq, France). The mobile phase was a gradient mixture of 0.1% formic acid (A) and acetonitrile (B). The flow rate was maintained at 1 mL/min, and the gradient profile was as follows: t0–11 min: A = 30% B = 70%; t11–15 min A = 70% B = 30%. The injection volume was 50 µL. The drug absorbance for quantification was obtained at 276 nm.

#### 2.3.3. Method Validation

The HPLC method was validated for specificity, the limit of detection (LOD), the limit of quantification (LOQ), linearity, precision, accuracy, according to ICH Q2 validation guidelines [18].

Linearity was determined by preparing one calibration curve daily for three days using five concentrations of ataluren at 50, 60, 70, 80, and 90 µg/mL, diluted in acetonitrile. For each calibration, the slope, intercept, and regression coefficient (r) were calculated as regression parameters by the least square method. ANOVA tests were applied to determine applicability. The accuracy for the active compound was determined by analyzing three replicates of samples prepared at 80%, 100%, and 120% of the target concentration. Accuracy was expressed as the percentage of recovery determined by experimental concentration/theoretical concentration × 100. The acceptance criterion was ± 2% deviation from the normal value for the recovery of ataluren. To verify the method’s precision, repeatability was estimated by calculating the relative standard deviation (RSD) of intraday analysis, and the intermediate precision was evaluated using RSD of inter-day analysis. Both RSDs should be less than 2%. For that, each day for three days, six solutions of ataluren 1% were prepared, analyzed, and quantified using a calibration curve prepared the same day. The limit of detection (LOD) and limit of quantification (LOQ) for ataluren assay were determined by calibration curve method using the following equations:

$$LOD=\;\frac{3.3\;x\;SD\;of\;y-intercept}{Slope\;of\;calibration\;curve};\;LOQ=\;\frac{10\;x\;SD\;of\;y-intercept}{Slope\;of\;calibration\;curve}$$

The matrix effect was evaluated by reproducing the previous methodology with the presence of all excipients present in the formulation and by comparing curves and intercepts.

The specificity was assessed by subjecting the ataluren 1% solubilized in DMSO (10%) and castor oil solutions to various forced degradation conditions: 0.1 M hydrochloric acid (HCl), 0.1 M sodium hydroxide (NaOH), 0.3% and 15% hydrogen peroxide (H_2_O_2_) for 24 h, 48 h, and 72 h at 60 °C. To mimic the potential photodegradation occurring prior to or after drug administration, photolytic studies were carried out by exposing the drug solutions to direct UV-visible light with and without photocatalyst or photosensitizer (i.e., titanium oxide 1 g L^−1^, riboflavin 100 mg L^−1^). Photolysis experiments were performed using a QSUN-XE-1 (Q-Lab, Bolton, UK) light chamber equipped with a xenon lamp, which simulates natural sunlight in a wavelength range of 300–800 nm. A Daylight-Q filter was used to simulate CIE Standard Illuminant D65 (Q-Lab), and the irradiance was maintained constant (1.5 W m^−2^ at 420 nm). The measurements corresponded to a visible intensity of ~119,600 lx and a UVA intensity at 300–400 nm of 66.5 W m^−2^. For all the experiments, the temperature was controlled and set at 25.0 ± 0.5 °C.

#### 2.3.4. Sterility Assay

Sterility is an absolute requirement of ophthalmic formulations. In order to evaluate the sterility of the eye drop in ophthalmic bottles, a test for sterility was carried out using the technique of membrane filtration with the product to be examined according to the European pharmacopeia [19].

To ensure applicability of the sterility test, sterility and fertility of media with and without the formulation were controlled. Six collection type strains were included corresponding to four bacteria (*Pseudomonas aeruginosa* ATCC 9027, *Staphylococcus aureus* ATCC 6538, *Clostridium sporogenes* ATCC 19404, and *Bacillus subtilis* ATCC 6633) and two fungi (*Candida albicans* ATCC 10231, *Aspergillus brasiliensis* ATCC 16404). Fluid thioglycollate medium and soya-bean casein digest medium were used as culture media. For each reference strain, 10 mL of formulations with and without the drug were filtered using the Steritest™ device (Steritest™ NEO, Merck Millipore, Guyancourt, France).

To validate the sterility applicability test, microbial growth clearly observable and visually comparable to that observed without product was observed each day for 5 days. To assess the sterility test of eye drops ataluren oily solution, the same procedure was applied, and the potential microbial growth was observed each day for 14 days.

### 2.4. Stability Study

Six bottles of the ataluren formulation were prepared and stored at 25 ± 3 °C. Physical and chemical examinations were performed in triplicate immediately after preparation (Day 0) and at Day 1, 3, 7, 14, 30, 60, and 90 to define drug stability throughout its period of storage except for the STAR ataluren suspension (Day 0, 7, and 21).

The chemical stability of the extemporaneous preparation was defined by the drug content that contained not less than 90% and not more than 110% of the labeled amount of ataluren [20].

### 2.5. Data Analysis-Acceptability Criteria

Data analyses were performed using Prism (GraphPad Software, version 7.04, San Diego, CA, USA, 2017). Descriptive statistics for continuous variables were expressed as mean ± SD.

The study was conducted following methodological guidelines issued by the International Conference on Harmonisation (ICH) for stability studies [18]. The instability of ataluren solutions was considered by a variation of concentration outside the 90–110% range of initial concentration of drug and the presence of degradation products. The observed solutions must be limpid, of unchanged color, and clear of visible signs of precipitation.

## 3. Results

### 3.1. Subsection

**Figure 1:**
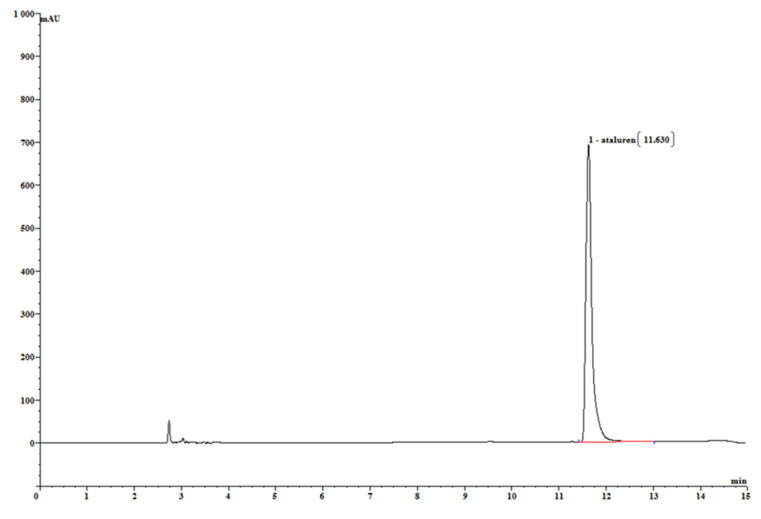
Reference chromatogram of ataluren at 276 nm in the oily solution.

The retention time of ataluren was observed to be about 11.6 min (Figure 1).

The chromatographic method used was found linear for concentrations ranging from 50 to 90 µg/mL. The calculated regression parameters are given in Table 1 and are within the linearity acceptance criteria. An average regression equation was y = 2.074 (± 0.017) x + 10.250 (± 1.271), where x is the ataluren concentration, and y is the surface area, and the average determination coefficient R^2^ of three calibration curves was 0.9995. No matrix effect was detected.

**Figure 2:**
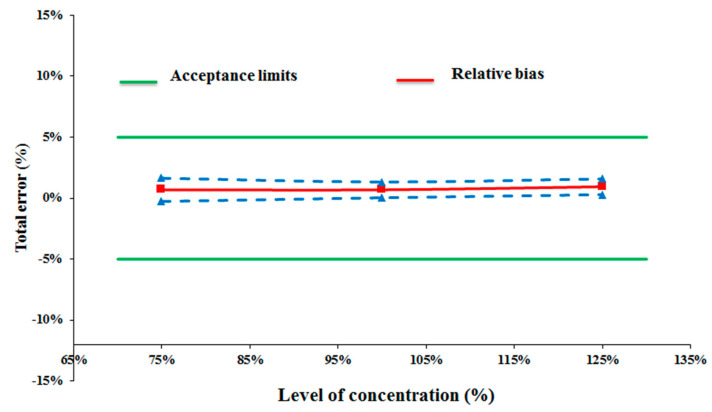
The 95% accuracy profile according to the ataluren level.

Results for intra-day precision and inter-day precision were less than 2.1%, as shown in Table 2. The 95% accuracy profile was within the predefined acceptance limits (Figure 2).

The determined values of LOD and LOQ were 6.8 µg/mL and 11.1 µg/mL, respectively, calculated using slope and Y-intercept.

When exposed to strong acidic, basic, or 0.3% H_2_O_2_ conditions, ataluren was not degraded after 7 days of exposure (Table 3).

**Figure 3:**
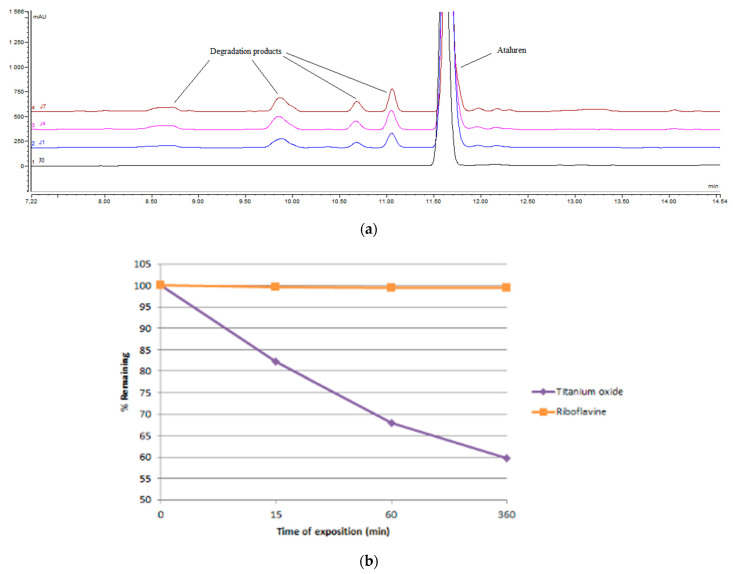
(**a**) Chromatograms of ataluren obtained at day 0 (black, control), 1 day (blue), 4 days (pink), and 7 days (orange) and its degradation products when exposed to 15% H_2_O_2_; (**b**) Indirect photolysis of ataluren.

Degradation products appeared only for 15% hydrogen peroxide (H_2_O_2_) exposure and are highlighted in Figure 3a.

In the direct photolytic stress condition, ataluren was not degraded (Table 4).

However, in the presence of a photocatalyst agent (titanium oxide), ataluren was rapidly degraded (Figure 3b). This was not the case when exposing the drug to light in the presence of riboflavin. Our method is stability-indicating as it enables separation between ataluren and its degradation products without peak interferences.

### 3.2. Chemical Stability of Ataluren Aqueous Suspension

The chemical stability of the STAR ataluren suspension (*n* = 6) showed a loss of the chemical stability greater than 10% at day 21 (Table 5).

### 3.3. Stability of Ataluren Oily Solution in Eyedroppers

#### 3.3.1. Physical Stability

There were no detectable visual changes in color and limpidity and no appearance of any visible particulate matter during the study period.

#### 3.3.2. Chemical Stability

The ataluren 1% eye drop oily solution stored in LDPE ophthalmic bottles at 22–25 °C demonstrated chemical stability for up to 60 days (Table 6). Ataluren retained at least 99% of its initial concentration at 60 days. Chromatograms showed no sign of degradation products throughout the study.

#### 3.3.3. Sterility Assay

The sterility applicability of the method was validated according to the European Pharmacopeia sterility assay [19]. The visual microbial growth in the control fertility experiments was clearly observed and comparable in the presence and absence of the product to be tested. No microbial growth was observed for any eye drop samples analyzed with this method over 14 days. Because the microbiological tests showed the absence of bacterial or fungal contamination of the preparation over time, the use of a preservative agent was not considered in the castor oil and DMSO eye drop formula.

## 4. Discussion

Our study reports new data on the stability of ataluren in ophthalmic solutions. The use of diverse co-solvent strategies (i.e., tween 80; cationorm^®^) only allowed partial ataluren solubilization, chemically stable less than 2–3 weeks. The use of castor oil and DMSO allowed us to obtain a 1% ataluren ophthalmic solution—exempt of suspended particles, sterile, and chemically stable. All parameters of the tested oily formulation were in favor of physicochemical and microbiological stability over 60 days.

Oxidation was shown critical in ataluren degradation, which is enhanced in aqueous media. The lack of water in the optimized formulation, combined with the use of pharmaceutical-grade castor oil controlled for its peroxide content, could also explain the enhanced stability of ataluren in the oily solution. In addition, the presence of DMSO, well-known for its antioxidant properties, may also have contributed to the absence of degradation perceived in the final formulation [21].

Simulated light experiments provided some insights into the propensity of ataluren to degrade both prior to and after administration. Ataluren concentration did not decrease upon direct light exposure, pointing out that the drug may resist to the light in the proposed formulation. Further, when riboflavin, a natural photosensitizing agent constituent of the eye, was added, no degradation was observed, which indicates that ataluren may not degrade through the photosensitized process naturally occurring in the eye [22]. Still, precaution should be taken as ataluren concentration decreased upon exposure when nanoparticles of titanium dioxide P25, a powerful photocatalyst, were added [23]. This points out that ataluren may degrade in the presence of a strong oxidizing agent, such as hydroxyl radical (•OH), which was detected in the context of nuclear cataract [24,25].

The sterility assay did not reveal any microbial contamination over the study time. The closure system eyedropper (Novelia^®^), which does not allow unfiltered air to penetrate the eyedropper, provided a further guarantee for sterility preservation of the content more than one month of simulated use [25]. Unlike a suspension, the sterilizing filtration process of removing microorganisms using a 0.22 µm filter can be performed with ataluren eye drops solution.

The ataluren eye drops concentration at 1% was selected according to a preclinical study showing its efficacy in a Pax6-deficient mouse model of aniridia [9]. In this study, the ataluren eye drop formulation was a suspension, which is less suitable than a solution in eye topical treatment. Indeed, some studies demonstrate the limitations of ophthalmic suspensions to deliver the proper dose after shaking because of the ineffective redispersion of particle sediment, also known as ‘cake’ or inappropriate manual shaking [26,27,28]. As suggested for steroids eye drops suspensions studies, some preclinical and clinical data show that reducing the drug particle size to the nanometer range in diameter provides effective ocular tissue penetration and more effective symptoms efficacy, even with reduced drug concentration and dosing frequency [29]. Ophthalmic suspensions are also known to cause ocular adverse effects, such as irritation, redness of the eye, and interference with vision [30]. Ataluren suspension caused marked ocular irritation, and the suspension formulation, referred to as START, abolished the irritation response seen with other formulations only apparently in mice [10].

DMSO is a drug characterized by anti-inflammatory, analgesic, and weak bacteriostatic properties and the ability to have radical scavenging activity [31,32]. It is also known as a well-tolerated penetration enhancing agent of drugs through biological membranes, including the cornea [33]. Based on these considerations, ophthalmic applications of DMSO have been already proposed and used in humans. Indeed, DMSO, known for its great solvating properties, is a well-known treatment for some eye diseases and is FDA-approved in some drug products like Onyx^®^ injection, Viadur^®^ implant, or Pennsaid^®^ topical gel [34,35,36]. DMSO, in association with various drugs, was safely used for ophthalmic applications in humans or animals [32,37,38]. Administration of up to 50% of DMSO in eye drops demonstrated the well acceptation of DMSO for ophthalmic use with no sign of irritation or toxicity [37,38,39,40,41,42,43]. In a recent study, the irritation potential and ocular tolerance of brinzolamide ophthalmic DMSO solution were evaluated when administered to the rabbit eye according to the OECD guidelines for acute eye irritation testing [43,44]. In all examined formulations of brinzolamide in DMSO for ophthalmic use, no sign of irritation or toxicity was observed [43]. In addition, DMSO has been used at 10–15% as a cryopreservation agent, safely used to freeze mammalian cells, including cornea, to guarantee cell viability after thawing [45]. Castor oil is a natural derivative of the Ricinus communis plant that possesses anti-inflammatory, anti-nociceptive, antioxidant, and antimicrobial properties [43,46,47,48]. Castor oil eye drops have been administered safely in several eye disorders like mild dry eye, blepharitis, contact lens discomfort, refractory Meibomian gland dysfunction [49,50,51].

## 5. Conclusions

In view of its physicochemical stability and its preservation of sterility, this study confirms 60 days stability of 1% ataluren eye drop solution when stored at 25 ± 3 °C in LDPE ophthalmic bottles. Ataluren eye drops aim to restore ocular surface PAX6 haploinsufficiency in congenital aniridia, and this new formulation opens to further clinical studies and innovative treatment for patients.

## Figures and Tables

**Table 1:** Linearity study of the stability-indicating analytic method.

Nominal Concentration (µg/mL)	Mean Peak Area ± SD (*n* = 45)	Calculated Amount (µg/mL)	Accuracy (%)
50	114.44 ± 0.76	50.2 ± 0.7	100.5
60	134.05 ± 0.81	59.7 ± 0.5	99.5
70	155.69 ± 0.79	70.1 ± 0.6	100.2
80	175.69 ± 1.50	79.8 ± 1.2	99.7
90	197.32 ± 0.63	90.2 ± 0.6	100.2

**Table 2:** Relative standard deviation values (%) for repeatability and intermediate precision (IQC). Definition: relative standard deviation (RSD).

Theoretical Concentration (µg/mL)	Mean Calculated Concentration (µg/mL)	% RSD Repeatability (*n* = 6)	% RSD Intermediate Precision (*n* = 3)
55	55.4 ± 0.6	0.57	0.79
70	70.5 ± 0.9	0.81	1.13
85	85.8 ± 1.8	1.46	2.02

**Table 3:** Forced degradation studies of 1% eye drop oily formulation.

Stress Conditions	% Remaining (Degradation)		
	Day 1	Day 3	Day 7
Acidic stress (0.1 M HCl, 60 °C)	100.1 (+0.1)	100.3 (+0.3)	100.4 (+0.4)
Alkaline stress (1 M NaOH, 60 °C)	100.2 (+0.2)	99.9 (−0.1)	100.1 (+0.1)
Oxidative stress (0.3% H_2_O_2_, 60 °C)	100.2 (+0.2)	99.2 (−0.8)	98.1 (−1.9)
Oxidative stress (15% H_2_O_2_, 60 °C)	99.4 (−0.6)	89.7 (−10.3)	74.1 (−25.9)

**Table 4:** Direct photolysis of ataluren. The measurements corresponded to a visible intensity of ~119,600 lx and an ultraviolet (UVA) intensity at 300–400 nm (66.5 W m^−2^).

Time of Exposition (min)	Mean Peak Area ± SD	% Remaining
30	240.2 ± 2.5	100.1
60	246.0 ± 1.9	102.4
180	240.4 ± 0.6	100.1
360	248.4 ± 2.1	103.4

**Table 5:** Chemical stability of the STAR ataluren suspension eye drops (0.9% Sodium chloride, 1% Tween 80, 1% Ataluren, 1% hydroxypropylcellulose) stored at 25 ± 3 °C (*n* = 3 in triplicate).

Bottles	Actual Concentration (100 mg/10 mL)	Mean ± SD% Ataluren Concentration Remaining	
	Day 0	Day 7	Day 21
A	100.4 ± 1.2	98.7 ± 1.1	84.0 ± 1.3
B	101.2 ± 1.5	97.9 ± 1.3	84.5 ± 1.4
C	99.8 ± 1.3	99.4 ± 1.1	84.0 ± 1.1

**Table 6:** Chemical stability of ataluren 1% eye drop castor oil and dimethylsulfoxide formulation stored at 22–25 °C in low-density polyethylene (LDPE) ophthalmic bottles and over time.

Eyedropper	Actual Concentration (100 mg/10 mL)	Mean ± SD% Ataluren Concentration Remaining				
	Day 0	Day 5	Day 15	Day 21	Day 30	Day 60
1	100.7 ± 1.3	99.7 ± 1.3	99.2 ± 1.2	99.2 ± 1.3	100.8 ± 1.2	100.9 ± 1.4
2	100.4 ± 1.7	97.8 ± 1.2	99.4 ± 1.4	99.4 ± 1.5	101.5 ± 1.5	101.0 ± 1.9
3	100.3 ± 1.5	99.4 ± 1.3	98.6 ± 1.5	99.0 ± 1.1	100.1 ± 1.2	100.9 ± 1.5

## Data Availability

Data is contained within the article.
